# Study on the Effect of Heavy Metal Contamination of Milk on the Coagulation Process

**DOI:** 10.3390/foods15091498

**Published:** 2026-04-25

**Authors:** Maria Natalia Chira, Sonia Amariei

**Affiliations:** Department of Food Engineering, Faculty of Food Engineering, Ștefan cel Mare University of Suceava, 720229 Suceava, Romania; cotosm@yahoo.co.uk

**Keywords:** heavy metals, controlled metal addition, milk coagulation, metal partitioning, lead, cadmium, copper

## Abstract

This study investigated how Pb, Cd, and Cu are distributed between curd and whey during milk coagulation in milk from different animal species, and how the level of metal addition and the coagulation method influence metal retention. Raw milk from buffalo, cow, donkey, goat, and sheep was supplemented with Pb, Cd, and Cu under controlled laboratory conditions at two levels corresponding to the regulatory maximum level (ML) and ten times this level (10 × ML). All three metals were added simultaneously to the same milk aliquot, and coagulation was induced either enzymatically or by acidification at pH 4.6. Metal concentrations in curd and whey were determined by atomic absorption spectrophotometry. In all milk types, Pb, Cd, and Cu were retained mainly in the curd fraction. At ML, curd retention generally ranged from about 77% to 97%, whereas at 10 × ML, retention decreased and transfer to whey increased. Donkey milk consistently showed lower metal retention in curd than ruminant milk. Statistical analysis of curd retention showed that metal type, milk species, the level of metal addition, and their interactions significantly influenced metal retention, indicating that the effect of coagulation method depended on the experimental conditions rather than being uniform across all cases. Overall, the results show that milk coagulation favours the association of Pb, Cd, and Cu with the curd fraction, highlighting the importance of the milk protein phase in determining metal distribution during dairy processing. These findings improve our understanding of heavy-metal behaviour during milk processing and help clarify their potential transfer into curd-based dairy products.

## 1. Introduction

Milk is a complex biological system with a highly organised colloidal structure composed of proteins, lipids, carbohydrates, and minerals. Because of this complexity, milk components can interact with a wide range of trace elements and metal ions. In particular, casein micelles, which represent the main protein structure in milk, contain multiple functional groups that bind metal ions via electrostatic and coordination interactions. As a result, both essential and potentially toxic metals may associate with milk constituents depending on their chemical form, concentration, and the physicochemical properties of the milk matrix [1,2,3].

Trace elements may enter milk through several environmental routes, including animal feed, soil, drinking water, and atmospheric deposition. Once absorbed by the animal, they may be transferred into milk during lactation. Several studies have reported the presence of heavy metals, including lead (Pb), cadmium (Cd), and copper (Cu), in raw milk from various geographic regions, underscoring the importance of continuous monitoring of contaminants in dairy systems [4,5,6,7,8]. Recent reviews have also emphasised that milk and dairy products may contribute to human exposure to toxic metals and metalloids, highlighting the relevance of analytical surveillance and risk assessment in dairy production [9].

Heavy metals are of particular concern in food systems because they are persistent and non-biodegradable and can accumulate in biological tissues. Chronic exposure can lead to significant adverse health effects. Lead has been linked to neurological and developmental toxicity, whereas cadmium is well known for its nephrotoxic potential and long-term accumulation in the body [10,11,12]. Copper is an essential trace element, but excessive intake may also be harmful because of its pro-oxidative effects and its capacity to disrupt cellular redox balance [10,11,12]. For this reason, regulatory frameworks such as Commission Regulation (EU) 2023/915 and Codex Alimentarius standards establish maximum permissible levels for contaminants in milk and dairy products [13,14].

Most studies on heavy metals in dairy systems have focused on measuring total metal concentrations in milk and estimating dietary exposure risks [6,7]. By contrast, much less attention has been paid to the behaviour of these metals during dairy processing. This is an important gap, because processing alters the distribution of milk components and may therefore change how metal ions partition within the system.

Milk coagulation is a key step in dairy processing, as it separates milk into two major fractions: the protein-rich curd and the aqueous whey phase. During coagulation, casein micelles aggregate to form a three-dimensional protein network that entraps fat globules and other components. Because metal ions can interact with proteins and minerals in milk, coagulation is expected to influence their distribution between curd and whey. In addition, differences in composition and coagulation behaviour among milk species may further affect the redistribution of metal ions during curd formation.

The affinity of metal ions for milk proteins is strongly linked to the structural properties of casein micelles. Casein molecules contain negatively charged functional groups, especially phosphoserine clusters and carboxylate residues, which can act as coordination sites for divalent metal ions [15,16]. These interactions may favour the incorporation of metal ions into the curd matrix during coagulation, thereby increasing their retention in curd-based dairy products [17,18]. Recent work on the milk colloidal phase has also shown that added metal-containing compounds can modify particle size distribution and ζ-potential through interactions with casein and micellar surface groups, further supporting the mechanistic relevance of protein–metal interactions in milk systems [19].

Despite the technological and toxicological relevance of this topic, systematic studies on heavy-metal partitioning during milk coagulation across different animal species remain limited. Differences in casein content, micellar structure, overall protein composition, and coagulation traits among species may influence the extent to which metals are retained in the curd fraction. Comparative studies on milk composition and coagulation traits in cow, goat, and sheep milk support this view and suggest that interspecies variation in milk structure and protein composition may contribute to differences in metal retention during curd formation [20].

Therefore, the aim of this study was to investigate how Pb, Cd, and Cu are distributed between curd and whey during milk coagulation in milk from buffalo, cow, donkey, goat, and sheep. Under controlled laboratory conditions, raw milk samples were supplemented with these metals at two levels corresponding to the regulatory maximum level (ML) and ten times that level (10 × ML). Both enzymatic and acid coagulation were applied, and the resulting curd and whey fractions were analysed by atomic absorption spectrophotometry to evaluate the distribution of added metals during milk coagulation.

## 2. Materials and Methods

### 2.1. Milk Sampling and Initial Characterisation

Raw milk samples were collected from dairy farms in three geographically distinct regions, each representing a different agro-industrial environment. Samples were obtained from five animal species: cow, goat, sheep, buffalo, and donkey. From each farm, at least 1 L of freshly produced raw milk was collected in a sterile container.

Immediately after collection, samples were refrigerated at 5 °C and transported to the laboratory, where they were processed the same day. Before experimental treatment, baseline concentrations of lead (Pb), cadmium (Cd), and copper (Cu) were determined in all samples to confirm the absence of quantifiable background metal levels. Basic physicochemical characteristics of the milk were also determined, including fat content (%), density (g mL^−1^), and total protein content (%). Milk fat was determined by the acido-butyrometric Gerber method according to SR ISO 19662:2023 [21]; total protein by the Kjeldahl method according to SR EN ISO 8968-1:2014 [22]; and density at 20 °C by the aerometric method using a calibrated thermolactodensimeter, in accordance with SR 2418:2008 [23] and SR 143:2008 [24]. These measurements were used to characterise milk composition and ensure comparability among species.

### 2.2. Reagents and Standards

All reagents used in this study were of analytical grade or higher purity. Nitric acid (65%; Merck KGaA, Darmstadt, Germany), hydrochloric acid (37%; Honeywell Specialty Chemicals Seelze GmbH, Seelze, Germany), orthophosphoric acid (85%; Merck KGaA, Darmstadt, Germany), glacial acetic acid (100%; Merck KGaA, Darmstadt, Germany), hydrogen peroxide (30%; Sigma-Aldrich, St. Louis, MO, USA), and ultrapure water (Carlo Erba Reagents S.A.S., Val-de-Reuil, France) were used throughout the analytical procedure. Certified single-element standard solutions were used for calibration and sample supplementation. The cadmium standard solution (1000 ± 3.3 mg L^−1^) was in the form of Cd(NO_3_)_2_ in 0.5 mol L^−1^ HNO_3_ (Merck KGaA, Darmstadt, Germany), and the lead standard solution (TraceCERT^®^ lead standard for AAS, 1000 ± 4 mg L^−1^ Pb) was in the form of Pb(NO_3_)_2_ in 2% HNO_3_ (Sigma-Aldrich Production GmbH, Buchs, Switzerland). The copper standard solution (1002 ± 5 mg L^−1^) was prepared from Cu(NO_3_)_2_·3H_2_O, was traceable to NIST SRM 3114, and was obtained from Agilent Technologies, North Kingstown, RI, USA. A multi-element ICP standard solution (50 mg L^−1^; Agilent ICM-120; Agilent Technologies, North Kingstown, RI, USA) was used to verify calibration. Certified reference material ERM^®^-BD151 skimmed milk powder (European Commission, Joint Research Centre, Geel, Belgium; Pb 0.207 ± 0.014 mg kg^−1^, Cd 0.106 ± 0.013 mg kg^−1^, and Cu 5.00 ± 0.23 mg kg^−1^) was used for quality control during analytical measurements. A mesophilic starter culture containing *Lactococcus lactis* subsp. *lactis*, *Lactococcus lactis* subsp. *cremoris*, and *Leuconostoc mesenteroides* subsp. *cremoris*, together with commercial rennet, was supplied by Browin, Sp. z o.o. Sp.k., Łódź, Poland.

### 2.3. Experimental Design and Sample Preparation

Milk samples obtained from independent farms were considered biological replicates. Before processing, the samples were brought to room temperature (approximately 22 °C) and gently mixed to ensure uniformity. For each biological replicate, milk was divided into 50 mL aliquots in centrifuge tubes. The experimental design included three sample groups: BLK, an unspiked control; ML, milk supplemented with metals at the regulatory maximum level; and 10 × ML, milk supplemented with metals at 10× the regulatory maximum level. Each group was subjected to two coagulation methods, enzymatic and acid, resulting in six conditions per biological replicate: BLK-enzymatic, BLK-acid, ML-enzymatic, ML-acid, 10 × ML-enzymatic, and 10 × ML-acid. The experiment was performed using three independent biological replicates for each milk species.

Metal addition was carried out by adding defined volumes of certified single-element standard solutions directly to 50 mL milk aliquots, followed by gentle mixing to ensure uniform distribution before coagulation. Pb, Cd, and Cu were added simultaneously rather than separately. The metals used were Pb(NO_3_)_2_ for lead, Cd(NO_3_)_2_ for cadmium, and Cu(NO_3_)_2_·3H_2_O for copper. After metal addition, the pH of each sample was readjusted to its initial value before coagulation.

Two levels of metal addition were prepared. In the ML group, Pb was adjusted to 0.020 mg kg^−1^ using a 10 mg L^−1^ standard solution (100 µL added to 50 mL milk), Cd to 0.010 mg kg^−1^ using a 10 mg L^−1^ standard solution (50 µL added to 50 mL milk), and Cu to 2.0 mg kg^−1^ using a 1000 mg L^−1^ standard solution (100 µL added to 50 mL milk). In the 10 × MLgroup, Pb was adjusted to 0.20 mg kg^−1^ using a 100 mg L^−1^ standard solution (100 µL added to 50 mL milk), Cd to 0.10 mg kg^−1^ using a 100 mg L^−1^ standard solution (50 µL added to 50 mL milk), and Cu to 20.0 mg kg^−1^ using a 1000 mg L^−1^ standard solution (1000 µL added to 50 mL milk). BLK samples received no metal addition and served as controls.

### 2.4. Coagulation Process

Following metal addition and pH readjustment to the initial value, the samples were gently homogenised and maintained at 22–25 °C for at least 1 h to allow equilibration and promote metal–milk interactions before coagulation. During this step, the samples were kept in a temperature-controlled shaking/incubation system (Titramax 1000 platform shaker with Incubator 1000, (Heidolph Instruments GmbH & Co. KG, Schwabach, Germany).

For enzymatic coagulation, the milk samples were heated to 32 °C, after which 50 mg of mesophilic starter culture and 100 µL of liquid rennet (activity 1:10,000 U) were added. The samples were then incubated at 32 °C for 1 h without agitation until coagulation was complete. For acid coagulation, the milk samples were heated to 32 °C, and a 1% (*v/v*) acetic acid solution was added slowly under gentle mixing until the pH reached 4.6. The samples were then incubated at 32 °C for 30 min to ensure complete coagulation.

After coagulation, the samples were centrifuged at 4 °C for 20 min at 4000 rpm using a refrigerated Harrier 18/80 centrifuge (MSE (UK) Ltd., London, UK)to separate the curd and whey fractions.

### 2.5. Sample Digestion for Metal Determination

Samples of curd and whey were prepared for metal analysis by microwave-assisted acid digestion. Approximately 0.5 g of each homogenised sample was accurately weighed into microwave digestion vessels. A mixture of 3.5 mL concentrated nitric acid (HNO_3_, 65%) and 1.5 mL hydrogen peroxide (H_2_O_2_, 30%) was added to each vessel to ensure complete decomposition of the organic matrix. All glassware and digestion vessels were acid-cleaned beforehand to minimise the risk of contamination. The digestion mixture was left to react overnight at room temperature and was then subjected to microwave digestion using a Speed Wave MWS-2 system (Berghof Products + Instruments GmbH, Eningen unter Achalm, Germany).

Microwave digestion was carried out using a three-step temperature programme at 80% microwave power: 145 °C for 10 min, followed by 160 °C for 10 min, and finally 190 °C for 20 min. After digestion, the vessels were allowed to cool to room temperature. The digested solutions were transferred to volumetric flasks and diluted to volume with ultrapure water before analysis.

### 2.6. Analytical Method and Validation

Lead (Pb) and cadmium (Cd) were determined by graphite furnace atomic absorption spectrophotometry (GFAAS), whereas copper (Cu) was determined by flame atomic absorption spectrophotometry (FAAS) using an air–acetylene flame. Measurements were performed on a Varian Duo AA280FS atomic absorption spectrometer coupled with a Varian AA280Z/GTA120/PSD120 graphite furnace system (Varian Australia Pty Ltd., Mulgrave, Victoria, Australia). Pyrolytically coated graphite tubes were used for GFAAS analysis, and background correction was performed using a deuterium lamp.

Quantification was based on external calibration with certified standard solutions. For Pb and Cd, calibration curves were prepared from standards in the form of Pb(NO_3_)_2_ and Cd(NO_3_)_2_. Calibration points were 0, 10, 20, 30, 40, and 50 µg L^−1^ for Pb, and 0, 1, 2, 3, 4, and 5 µg L^−1^ for Cd. Copper was measured using an Agilent hollow cathode lamp (lamp code 5610123300) at 324.8 nm, and the calibration curve was established at 0.0, 0.2, 0.4, 0.6, 0.8, and 1.0 mg L^−1^ using a certified standard prepared from Cu(NO_3_)_2_·3H_2_O. A multi-element ICP standard solution (Agilent ICM-120, 50 mg L^−1^) was used for calibration verification.

To minimise matrix interference during GFAAS analysis, a 3% (*w*/*v*) orthophosphoric acid solution served as a chemical modifier. Measurements were made at 283.3 nm for Pb, 228.8 nm for Cd, and 324.8 nm for Cu. The calibration curves showed excellent linearity, with R^2^ values greater than 0.999. The limits of detection (LODs) were 0.003 mg kg^−1^ for Pb, 0.0006 mg kg^−1^ for Cd, and 0.03 mg kg^−1^ for Cu, while the limits of quantification (LOQs) were 0.010, 0.002, and 0.1 mg kg^−1^, respectively.

All measurements were performed in triplicate. Method accuracy was verified using the certified reference material ERM^®^-BD151 skimmed milk powder (Pb 0.207 ± 0.014 mg kg^−1^, Cd 0.106 ± 0.013 mg kg^−1^, and Cu 5.00 ± 0.23 mg kg^−1^). Procedural blanks and quality-control samples were included in each analytical batch. Recovery rates ranged from 92% to 105% for Pb, 90% to 103% for Cd, and 94% to 107% for Cu, confirming acceptable method accuracy. Repeatability, assessed under identical conditions, yielded relative standard deviations (RSDs) below 5% for all metals. Metal concentrations in the samples were calculated using the following equation: w = (a × V × F)/m, where w is the metal concentration in the sample (mg kg^−1^), a is the concentration measured in the analytical solution (µg L^−1^), V is the final solution volume (mL), F is the dilution factor, and m is the initial sample mass (g).

### 2.7. Statistical Analysis

Results are presented as mean ± standard deviation based on three independent biological replicates (*n* = 3) for each milk species. Biological replicates consisted of milk samples obtained from different farms and processed separately throughout the experimental procedure. Statistical analyses were performed in R software (version 4.5.2; R Foundation for Statistical Computing, Vienna, Austria).

BLK samples were used only as unspiked negative controls to confirm the absence of quantifiable background metal levels and were not included in the inferential statistical analysis. Because whey retention is the algebraic complement of curd retention, and the partition coefficient (K) is a deterministic transformation of these values, inferential modelling focused on metal retention in the curd fraction.

Curd retention proportions were analysed using a full-factorial mixed-effects model with beta error distribution and logit link. Milk species, level of metal addition, coagulation method, and metal type were included as fixed effects together with their interactions. A biological replicate was included as a random effect, and aliquot identity nested within biological replicate was included as an additional random intercept to account for non-independence among measurements obtained from the same milk sample under the same treatment condition. The significance of fixed effects and their interactions was assessed using Type III Wald chi-square tests. Model diagnostics were evaluated using simulation-based residual analysis.

When significant effects were identified, post hoc comparisons were performed using estimated marginal means. Tukey-adjusted comparisons were used to compare milk species within the same metal × level of metal addition × coagulation method combination. In contrast, Holm-adjusted contrasts were used for targeted comparisons of coagulation method, metal addition level, and metal type. Differences were considered statistically significant at *p* < 0.05.

The partition coefficient (K), calculated as the ratio between metal retention in the curd fraction and metal retention in the whey fraction, was retained as a descriptive indicator of curd-versus-whey partitioning and is presented in Supplementary Table S1.

## 3. Results

### 3.1. Initial Composition of Raw Milk Samples

Raw milk samples from all investigated species showed Pb, Cd, and Cu concentrations below the limit of quantification (LOQ), indicating no quantifiable background contamination and supporting their use as control samples (BLK). The main physicochemical parameters, including fat content, density, and total protein, were within the expected ranges for each species (Table 1). Clear interspecies differences were observed, particularly in fat and total protein content, which may contribute to differences in metal partitioning during coagulation and are discussed further.

### 3.2. Distribution of Heavy Metals Between Curd and Whey Fractions

Milk coagulation caused a clear redistribution of Pb, Cd, and Cu between the curd and whey fractions, with most of each metal remaining in the curd phase across all experimental conditions (Table 2). At the ML level, curd retention ranged from 77.56% to 96.64%. Pb showed high retention in the curd across all milk species, although donkey milk had the lowest values under both acid coagulation (77.56%) and enzymatic coagulation (81.57%). For Cd, curd retention at the ML level remained above 87% in all cases, with lower values in goat and sheep milk than in buffalo and cow milk. Cu showed the highest overall retention, reaching 96.64% in buffalo milk under acid coagulation.

At the 10 × MLlevel, curd retention decreased for all three metals, indicating a corresponding increase in transfer to the whey fraction. This trend was especially evident for Pb in donkey milk, where curd retention decreased to 74.96% under acid coagulation and 72.56% under enzymatic coagulation. For Cd, lower retention values at 10 × MLwere particularly observed in sheep and goat milk, whereas buffalo milk maintained the highest curd retention. Cu also showed a noticeable decrease in retention at the higher metal-addition level across several species, particularly in cow, sheep, and donkey milk.

Overall, these results show that the distribution of Pb, Cd, and Cu during coagulation depended on metal type, milk species, metal addition level, and coagulation method. As shown in Table 3, the statistical analysis identified significant interaction effects among these factors, indicating that the influence of coagulation method was condition-dependent rather than uniform across all experimental combinations.

### 3.3. Comparative Analysis of Partitioning Behaviour of Pb, Cd, and Cu and the Influence of the Level of Metal Addition

**Figure 1:**
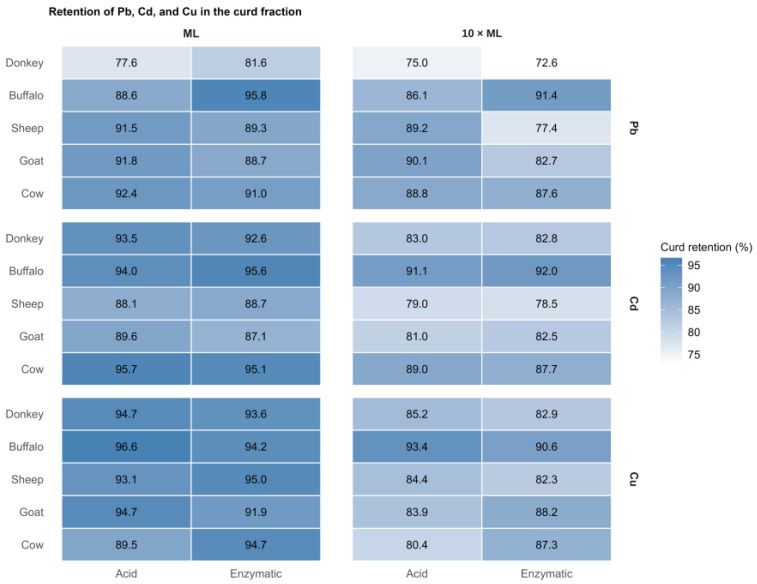
Heatmap showing the mean retention (%) of Pb, Cd, and Cu in the curd fraction according to milk species, level of metal addition (ML and 10 × ML), and coagulation method (acid or enzymatic). Each cell represents the mean of three independent biological replicates.

To compare the retention behaviour of the investigated metals across all experimental conditions, the distribution patterns of Pb, Cd, and Cu in the curd fraction were examined simultaneously using a comparative heatmap (Figure 1). The heatmap provides an integrated overview of curd retention as affected by milk species, coagulation method, metal type, and metal addition level. It highlights both the general tendency of these metals to remain in the curd fraction and the condition-specific differences observed among treatments.

Across all treatments, Pb, Cd, and Cu showed a clear tendency to be preferentially retained in the curd fraction. However, the extent of retention varied among metals and experimental conditions. In general, higher metal addition levels were associated with lower relative retention in the curd and, consequently, greater transfer to the whey fraction. However, the overall direction of partitioning remained unchanged.

The partition coefficient (K), calculated as the ratio of metal retention in curd to that in whey, was used as a descriptive indicator of curd-versus-whey partitioning and is presented in Supplementary Table S1. Across all experimental conditions, K values remained greater than 1, confirming the preferential association of Pb, Cd, and Cu with the curd fraction. However, because K is a derived measure based on curd and whey retention, inferential analysis focused on curd retention as the primary response variable.

These trends were consistent with the mixed-effects analysis of curd retention presented in Table 3. Although the main effect of coagulation method was not significant when considered in isolation (*p* = 0.0757), several interaction terms involving coagulation method were significant, including the four-way interaction among milk species, level of metal addition, coagulation method, and metal type (*p* < 0.001). This indicates that the effect of the coagulation method depended on the specific experimental context. Taken together, these results show that heavy-metal partitioning during milk coagulation was governed by a combination of metal type, milk species, metal addition level, and coagulation method, rather than by any single factor acting independently.

## 4. Discussion

Although all examined milk types had Pb, Cd, and Cu concentrations below the limit of quantification before metal addition, compositional differences among species remain important for understanding metal partitioning during coagulation. Variations in total protein and casein content, micellar structure, and fat-to-protein ratio provide a plausible structural basis for species-dependent differences in metal distribution between curd and whey. This is particularly relevant for donkey milk, which contains substantially lower total protein and casein levels than ruminant milk and therefore likely offers fewer coordination sites for divalent metal ions. This interpretation aligns with comparative studies showing marked differences in milk composition and coagulation traits among cow, goat, and sheep milk, supporting the view that interspecies variation in protein composition and coagulation behaviour can influence metal retention during curd formation [15,16,20].

The absence of measurable background metal levels enabled the redistribution of added metals to be assessed under controlled laboratory conditions. This is important because naturally contaminated milk may contain metals in different physicochemical forms, depending on their origin and prior association with milk constituents. Under the present design, the observed partitioning behaviour can therefore be interpreted primarily in relation to the intrinsic properties of the metal ions, the characteristics of the milk system, and the coagulation process itself.

The preferential accumulation of Pb, Cd, and Cu in the curd fraction aligns with the known chemistry of casein micelles. Casein proteins contain multiple negatively charged functional groups, particularly phosphoserine clusters and carboxylate residues, which can serve as coordination sites for divalent metal ions [15,16]. During coagulation, aggregation of casein micelles promotes the incorporation of these ions into the developing protein network, thereby favouring retention in the curd rather than in the aqueous whey phase. Similar behaviour has been reported in studies of metal distribution in dairy systems and processed milk products [17,18,25]. Recent work on the milk colloidal phase has also shown that added metal-containing compounds can alter physicochemical properties, such as particle size distribution and ζ-potential, through interactions with casein and micellar surface groups. Although that work focused on Zn rather than Pb, Cd, or Cu, it supports the broader mechanistic idea that metal ions can modify the behaviour of the milk protein phase by coordinating to negatively charged sites [19].

The role of the coagulation method should also be considered from a physicochemical perspective. In acid coagulation, lowering the pH to 4.6 alters the ionisation state of casein functional groups, destabilising the colloidal calcium phosphate–casein micellar system. These changes can influence both the number and strength of available metal-binding sites and may also affect the distribution of soluble and colloidal mineral species between curd and whey. By contrast, enzymatic coagulation primarily destabilises the micellar surface by cleaving κ-casein while maintaining a different ionic environment. Recent work comparing coagulants has shown that the nature of the coagulating agent can affect coagulation dynamics and curd development, even when the final technological outcome remains broadly similar [26]. However, in the present study, the influence of the coagulation method was not uniform across the dataset. Instead, the statistical analysis showed that its effect depended on the specific combination of milk species, metal type, and level of metal addition, which is consistent with the significant interaction terms observed in the mixed-effects model.

The interspecies differences observed here are also consistent with known variation in milk composition. Buffalo and sheep milk are richer in total solids and protein than donkey milk, whereas cow and goat milk occupy an intermediate position. These compositional differences are likely to influence the density of available binding sites for Pb^2+^, Cd^2+^, and Cu^2+^ and may also affect curd structure and water retention. In the present study, donkey milk consistently exhibited lower metal retention in the curd fraction, which aligns with its lower protein content and distinct casein profile. Therefore, although the overall direction of partitioning was similar across species, the extent of retention clearly depended on the milk matrix.

The present findings are also broadly consistent with previous reports showing preferential transfer of heavy metals to the curd or cheese fraction during dairy processing. In a recent study of buffalo milk, Grassi and Perna examined Pb- and Cd-containing milk processed into Caciotta cheese and found that, although the gross chemical composition was not significantly altered, oxidative stability markers in the cheese matrix were adversely affected [26]. Although that study focused on the quality of the final cheese rather than on curd–whey partitioning itself, it supports the technological relevance of metal retention in the protein-rich dairy fraction, reinforcing the food-safety implications of the present results.

The level of metal addition influenced the extent, but not the direction, of metal partitioning. Increasing the level from ML to 10 × MLgenerally reduced relative retention in the curd and increased transfer to whey. However, these data do not, by themselves, demonstrate saturation of binding sites in a strict mechanistic sense, because no binding isotherm or concentration–response series was established. A more cautious interpretation is that higher metal loading reduced the curd’s relative retention capacity, which is consistent with a finite binding capacity of the casein-rich matrix and/or mass-action-driven redistribution towards the whey fraction. The decrease in the partition coefficient (K) at higher metal addition levels is consistent with this interpretation, but should not be taken as direct proof of site saturation. In the revised manuscript, K is retained only as a descriptive indicator and is presented in Supplementary Table S1.

The statistical analysis supports this overall interpretation, but it also shows that the system is more complex than a simple main-effects model would suggest. Metal type, milk species, and the level of metal addition were all important contributors to curd retention, but their effects were strongly modified by interaction terms involving coagulation method. Therefore, partitioning during coagulation cannot be attributed to a single dominant factor acting independently; rather, it reflects the combined influence of metal chemistry, milk composition, the level of metal addition, and coagulation conditions.

A limitation of the present study is that analytical validation relied on a bovine skimmed-milk certified reference material. In contrast, the experimental design also included buffalo, goat, sheep, and donkey milk. Therefore, species-specific matrix effects cannot be entirely ruled out. In addition, only two levels of metal addition were evaluated, and no binding isotherm or kinetic analysis was performed. Future studies should include a wider concentration range, matrix-matched validation across species, and direct comparison with pilot-scale cheese-making systems to better understand the mechanisms controlling metal retention in dairy systems.

## 5. Conclusions

This study showed that Pb, Cd, and Cu were preferentially retained in the curd fraction during milk coagulation across all tested milk species. Although the overall direction of partitioning was similar across milk types, the extent of retention varied by species, with donkey milk generally showing lower metal retention in the curd than ruminant milk. The level of metal addition also influenced metal distribution: increasing metal addition from ML to 10 × MLreduced relative retention in the curd and increased transfer to the whey fraction.

The statistical analysis confirmed that curd retention was influenced by metal type, milk species, level of metal addition, and their interactions, indicating that the effect of coagulation method depended on the specific experimental conditions rather than acting uniformly across all cases. These findings suggest that metal partitioning during coagulation is governed by a combination of metal chemistry, milk composition, level of metal addition, and coagulation conditions.

From a mechanistic perspective, the preferential accumulation of metals in the curd fraction is consistent with interactions between divalent metal ions and negatively charged groups in casein micelles, particularly phosphoserine clusters and carboxylate residues. However, the lower partition coefficients observed at higher levels of metal addition should be interpreted with caution, as they indicate reduced relative retention in the curd phase but, by themselves, do not demonstrate saturation of binding sites.

Overall, the results highlight the food-safety relevance of metal partitioning during dairy processing, since metals present in raw milk may be preferentially transferred to curd-based products. Future studies should include a broader range of metal addition levels, species-specific matrix validation, and pilot-scale cheese-making conditions to better understand the mechanisms that control metal retention in dairy systems.

## Figures and Tables

**Table 1:** Initial composition of raw milk from all investigated species.

Species	Pb (mg kg^−1^)	Cd (mg kg^−1^)	Cu (mg kg^−1^)	Fat (%)	Density (g mL^−1^)	Total Protein (%)
Cow	<LOQ	<LOQ	<LOQ	3.72 ± 0.24	1.030 ± 0.001	3.36 ± 0.15
Goat	<LOQ	<LOQ	<LOQ	3.84 ± 0.25	1.029 ± 0.001	3.48 ± 0.16
Sheep	<LOQ	<LOQ	<LOQ	7.56 ± 0.49	1.033 ± 0.001	6.93 ± 0.32
Buffalo	<LOQ	<LOQ	<LOQ	8.11 ± 0.52	1.031 ± 0.001	4.59 ± 0.21
Donkey	<LOQ	<LOQ	<LOQ	0.51 ± 0.033	1.032 ± 0.001	1.89 ± 0.084

Note: Values are expressed as mean ± standard deviation (SD) of three independent biological replicates (n = 3). LOQ values were: Pb = 0.010 mg kg^−1^, Cd = 0.002 mg kg^−1^, Cu = 0.1 mg kg^−1^.

**Table 2:** Retention (%) of Pb, Cd, and Cu in the curd fraction obtained from milk of different species after acid or enzymatic coagulation at two levels of metal addition (ML and 10 × ML).

Level of Metal Addition: ML			
Metal	Species	Acid	Enzymatic
Pb	Cow	92.40 ± 1.49 ^c^	91.01 ± 1.68 ^c^
Pb	Goat	91.83 ± 0.39 ^c^	88.74 ± 0.34 ^b^
Pb	Sheep	91.53 ± 0.47 ^c^	89.28 ± 0.61 ^bc^
Pb	Buffalo	88.57 ± 0.24 ^b^	95.82 ± 0.08 ^d^
Pb	Donkey	77.56 ± 0.11 ^a^	81.57 ± 0.36 ^a^
Cd	Cow	95.74 ± 1.58 ^c^	95.07 ± 1.73 ^c^
Cd	Goat	89.58 ± 0.93 ^a^	87.07 ± 0.85 ^a^
Cd	Sheep	88.14 ± 0.48 ^a^	88.70 ± 0.14 ^a^
Cd	Buffalo	93.98 ± 1.45 ^b^	95.64 ± 0.37 ^c^
Cd	Donkey	93.52 ± 0.57 ^b^	92.61 ± 0.32 ^b^
Cu	Cow	89.49 ± 0.09 ^a^	94.75 ± 1.52 ^b^
Cu	Goat	94.73 ± 0.40 ^c^	91.89 ± 0.65 ^a^
Cu	Sheep	93.11 ± 0.56 ^b^	95.01 ± 0.61 ^b^
Cu	Buffalo	96.64 ± 0.36 ^d^	94.22 ± 0.88 ^b^
Cu	Donkey	94.72 ± 0.66 ^c^	93.64 ± 0.11 ^b^
**Level of Metal Addition: 10 × ML**			
**Metal**	**Species**	**Acid**	**Enzymatic**
Pb	Cow	88.80 ± 1.18 ^c^	87.62 ± 1.49 ^d^
Pb	Goat	90.05 ± 0.50 ^c^	82.69 ± 0.88 ^c^
Pb	Sheep	89.25 ± 0.48 ^c^	77.37 ± 0.31 ^b^
Pb	Buffalo	86.13 ± 0.52 ^b^	91.43 ± 0.64 ^e^
Pb	Donkey	74.96 ± 0.16 ^a^	72.56 ± 0.12 ^a^
Cd	Cow	88.97 ± 1.95 ^c^	87.74 ± 2.34 ^c^
Cd	Goat	81.03 ± 0.23 ^ab^	82.52 ± 0.28 ^b^
Cd	Sheep	78.96 ± 0.42 ^a^	78.50 ± 0.50 ^a^
Cd	Buffalo	91.13 ± 0.63 ^d^	92.01 ± 0.14 ^d^
Cd	Donkey	83.04 ± 0.47 ^b^	82.76 ± 0.08 ^b^
Cu	Cow	80.36 ± 1.72 ^a^	87.27 ± 2.92 ^b^
Cu	Goat	83.93 ± 1.32 ^b^	88.23 ± 1.23 ^b^
Cu	Sheep	84.42 ± 0.28 ^b^	82.27 ± 0.47 ^a^
Cu	Buffalo	93.37 ± 1.09 ^c^	90.57 ± 0.31 ^c^
Cu	Donkey	85.19 ± 0.27 ^b^	82.92 ± 0.75 ^a^

Note: Values are expressed as mean ± standard deviation (n = 3). Different superscript letters within the same metal × level of metal addition × coagulation method indicate significant differences among milk species according to Tukey-adjusted post hoc comparisons (*p* < 0.05).

**Table 3:** Type III analysis of deviance for the effects of milk species, level of metal addition, coagulation method, metal type, and their interactions on heavy-metal retention in the curd fraction.

Effect	Df	Chi-Square	*p*-Value
Milk species	4	789.708	<0.001
Level of metal addition	1	2565.541	<0.001
Coagulation method	1	3.155	0.0757
Metal type	2	443.272	<0.001
Milk species × Level of metal addition	4	76.666	<0.001
Milk species × Coagulation method	4	112.401	<0.001
Level of metal addition × Coagulation method	1	8.915	0.0028
Milk species × Metal type	8	1424.002	<0.001
Level of metal addition × Metal type	2	229.804	<0.001
Coagulation method × Metal type	2	5.866	0.0532
Milk species × Level of metal addition × Coagulation method	4	76.310	<0.001
Milk species × Level of metal addition × Metal type	8	104.399	<0.001
Milk species × Coagulation method × Metal type	8	517.110	<0.001
Level of metal addition × Coagulation method × Metal type	2	50.930	<0.001
Milk species × Level of metal addition × Coagulation method × Metal type	8	61.685	<0.001

Note: The significance of fixed effects and their interactions was assessed using Type III Wald chi-square tests from the full mixed-effects model fitted to the curd retention data. BLK samples were used only as negative controls and were not included in the inferential analysis. Because a significant four-way interaction was detected, lower-order terms should be interpreted in the context of the overall interaction structure.

## Data Availability

The original contributions presented in this study are included in the article/Supplementary Material. Further inquiries can be directed to the corresponding author.

## Supplementary Materials

The following supporting information can be downloaded at https://www.mdpi.com/article/10.3390/foods15091498/s1. Table S1. Partition coefficient (K = curd/whey) for Pb, Cd, and Cu according to milk species, level of metal addition (ML and 10 × ML), and coagulation method (acid or enzymatic).

## Abbreviations

The following abbreviations are used in this manuscript:

AAS	Atomic absorption spectrophotometry
GFAAS	Graphite furnace atomic absorption spectrophotometry
FAAS	Flame atomic absorption spectrophotometry
LOD	Limit of detection
RSD	Relative standard deviation
ML	Maximum level
BLK	Blank sample
K	Partition coefficient
