# Supplementary material for: Study on the Effect of Heavy Metal Contamination of Milk on the Coagulation Process
Source: Foods. 2026 Apr 25;15(9):1498. doi: 10.3390/foods15091498 (PMC13164320; doi:10.3390/foods15091498)
Supplement: Supplementary file 1 [file foods-15-01498-s001.zip › foods-4241117-supplementary.pdf]

Table S1. Partition coefficient (K = curd/whey) for Pb, Cd, and Cu according to milk species, level of metal addition (ML and 10×ML), and coagulation method (acid or enzymatic).

Level of metal addition: ML

Metal	Species	Acid	Enzymatic
Cd	Buffalo	16.314 ± 4.394	22.035 ± 1.968
Cd	Cow	25.264 ± 11.419	21.309 ± 8.850
Cd	Donkey	14.497 ± 1.294	12.549 ± 0.578
Cd	Goat	8.649 ± 0.856	6.754 ± 0.513
Cd	Sheep	7.439 ± 0.345	7.854 ± 0.109
Cu	Buffalo	28.960 ± 3.363	16.613 ± 2.955
Cu	Cow	8.518 ± 0.079	19.100 ± 5.533
Cu	Donkey	18.168 ± 2.581	14.715 ± 0.264
Cu	Goat	18.053 ± 1.461	11.382 ± 0.991
Cu	Sheep	13.586 ± 1.239	19.239 ± 2.618
Pb	Buffalo	7.751 ± 0.184	22.942 ± 0.443
Pb	Cow	12.538 ± 2.930	10.396 ± 2.244
Pb	Donkey	3.457 ± 0.023	4.427 ± 0.108
Pb	Goat	11.259 ± 0.602	7.887 ± 0.271
Pb	Sheep	10.837 ± 0.650	8.346 ± 0.535

Level of metal addition: 10×ML

Metal	Species	Acid	Enzymatic
Cd	Buffalo	10.312 ± 0.812	11.523 ± 0.220
Cd	Cow	8.245 ± 1.504	7.342 ± 1.464
Cd	Donkey	4.898 ± 0.165	4.799 ± 0.028
Cd	Goat	4.273 ± 0.065	4.723 ± 0.091
Cd	Sheep	3.755 ± 0.095	3.653 ± 0.110
Cu	Buffalo	14.337 ± 2.336	9.609 ± 0.340
Cu	Cow	4.117 ± 0.426	7.127 ± 1.792
Cu	Donkey	5.752 ± 0.122	4.861 ± 0.254
Cu	Goat	5.254 ± 0.530	7.562 ± 0.934
Cu	Sheep	5.422 ± 0.114	4.644 ± 0.148
Pb	Buffalo	6.217 ± 0.266	10.719 ± 0.914
Pb	Cow	7.991 ± 0.892	7.150 ± 0.925
Pb	Donkey	2.993 ± 0.026	2.644 ± 0.016
Pb	Goat	9.069 ± 0.502	4.787 ± 0.301
Pb	Sheep	8.314 ± 0.403	3.420 ± 0.061

Note: Values are expressed as mean ± standard deviation (n = 3). The partition coefficient K was calculated as the ratio between metal retention in the curd fraction and metal retention in the whey fraction. K values are presented as a descriptive indicator of curd-versus-whey partitioning and were not used as an independent inferential response variable.
